# Association Between 5-Year Clinical Outcome in Patients With Nonmedically Evacuated Mild Blast Traumatic Brain Injury and Clinical Measures Collected Within 7 Days Postinjury in Combat

**DOI:** 10.1001/jamanetworkopen.2018.6676

**Published:** 2019-01-04

**Authors:** Christine L. Mac Donald, Jason Barber, Jana Patterson, Ann M. Johnson, Sureyya Dikmen, Jesse R. Fann, Nancy Temkin

**Affiliations:** 1 Department of Neurological Surgery, University of Washington, Seattle; 2 Center for Clinical Studies, Washington University, Saint Louis Missouri; 3 Department of Rehabilitation Medicine, University of Washington, Seattle; 4 Department of Psychiatry, University of Washington, Seattle; 5 Department of Biostatistics, University of Washington, Seattle

## Abstract

**Importance:**

Although previous work has examined clinical outcomes in combat-deployed veterans, questions remain regarding how symptoms evolve or resolve following mild blast traumatic brain injury (TBI) treated in theater and their association with long-term outcomes.

**Objective:**

To characterize 5-year outcome in patients with nonmedically evacuated blast concussion compared with combat-deployed controls and understand what clinical measures collected acutely in theater are associated with 5-year outcome.

**Design, Setting, and Participants:**

A prospective, longitudinal cohort study including 45 service members with mild blast TBI within 7 days of injury (mean 4 days) and 45 combat deployed nonconcussed controls was carried out. Enrollment occurred in Afghanistan at the point of injury with evaluation of 5-year outcome in the United States. The enrollment occurred from March to September 2012 with 5-year follow up completed from April 2017 to May 2018. Data analysis was completed from June to July 2018.

**Exposures:**

Concussive blast TBI. All patients were treated in theater, and none required medical evacuation.

**Main Outcomes and Measures:**

Clinical measures collected in theater included measures for concussion symptoms, posttraumatic stress disorder (PTSD) symptoms, depression symptoms, balance performance, combat exposure intensity, cognitive performance, and demographics. Five-year outcome evaluation included measures for global disability, neurobehavioral impairment, PTSD symptoms, depression symptoms, and 10 domains of cognitive function. Forward selection multivariate regression was used to determine predictors of 5-year outcome for global disability, neurobehavior impairment, PTSD, and cognitive function.

**Results:**

Nonmedically evacuated patients with concussive blast injury (n = 45; 44 men, mean [SD] age, 31 [5] years) fared poorly at 5-year follow-up compared with combat-deployed controls (n = 45; 35 men; mean [SD] age, 34 [7] years) on global disability, neurobehavioral impairment, and psychiatric symptoms, whereas cognitive changes were unremarkable. Acute predictors of 5-year outcome consistently identified TBI diagnosis with contribution from acute concussion and mental health symptoms and select measures of cognitive performance depending on the model for 5-year global disability (area under the curve following bootstrap validation [AUC_BV_] = 0.79), neurobehavioral impairment (correlation following bootstrap validation [R_BV_] = 0.60), PTSD severity (R_BV_ = 0.36), or cognitive performance (R_BV_ = 0.34).

**Conclusions and Relevance:**

Service members with concussive blast injuries fared poorly at 5-year outcome. The results support a more focused acute screening of mental health following TBI diagnosis as strong indicators of poor long-term outcome. This extends prior work examining outcome in patients with concussive blast injury to the larger nonmedically evacuated population.

## Key Points

**Question:**

What clinical measures collected acutely in combat are associated with 5-year outcome in patients with concussive blast injury?

**Findings:**

In this longitudinal cohort study, nonmedically evacuated blast concussion patients had significant and sustained symptoms of neurobehavioral impairment, mental health and global disability, whereas cognitive changes were unremarkable compared with combat-deployed nonconcussed controls. Assessments collected in theater were associated with multiple domains of outcome.

**Meaning:**

Nonmedically evacuated patients with concussive blast injury, considered the mildest of the mild combat casualties fared poorly 5 years later compared with combat-deployed controls.

## Teaser

This cohort study characterizes 5-year outcome in nonmedically evacuated blast concussion patients compared with combat-deployed controls to evaluate what clinical measures collected acutely in theater are associated with 5-year outcomes.

## Introduction

The long-term clinical impact of war-time mild blast-related traumatic brain injury (TBI) remains incompletely described.^1,2^ Previous studies have been based largely on self-report and screening tools^3,4,5,6^ to define TBI, rather than direct clinical assessments in cohorts identified at the time of injury and prospectively studied. Although much effort has been expended to better understand this type of concussive TBI, many studies in active-duty US military and veterans have been restricted to cross-sectional evaluations,^4,7,8,9,10,11,12,13,14,15^ often involving retrospective record review^7,8,9,16^ or self-report,^4,6,10,11,12,14,16,17,18,19^ and considering only chronic phases of injury.^13,16,18,20,21^

Few longitudinal studies have been completed in this population, largely restricted to the first year after exposure^19,22,23,24,25,26^ or by serial evaluation only in the chronic stage.^27,28^ One prior study compared predeployment, postdeployment, and more than 5-year follow-up; however, the study did not restrict inclusion to just mild TBI and did not discriminate between medically evacuated vs nonmedically evacuated cases.^29^ The findings and prior body of literature motivate further research to better characterize risk factors that can be associated with long-term outcomes after specifically mild TBI exposures in combat. Questions remain regarding how symptoms evolve or resolve following mild blast-related TBI treated in theater and how they are associated with the service member’s long-term trajectory.

Our own work has shown an evolution, not resolution, of symptoms by 5-year follow-up in patients with blast-related mild TBI^30,31^ who were medically evacuated from the combat theater. Less is known about the long-term outcome trajectory in the larger population of nonmedically evacuated service members who sustain blast-related mild TBI, mild enough to remain in theater for treatment and return to their unit. Through collaborative efforts at Kandahar Airfield, Camp Leatherneck, and academic universities in the United States, we have been provided the unique opportunity to follow the very same patients from the point of injury in theater^32^ to both 1-year,^22^ and now 5-year outcome. The objective of the current study was to characterize 5-year outcome in patients with nonmedically evacuated mild blast TBI and to understand what clinical measures collected within the first week of injury in combat best predicted clinical outcome 5 years later because this has important implications for acute care considerations of combat casualties who have sustained these concussion exposures.

## Methods

Participants were initially enrolled at Kandahar Air Field and Camp Leatherneck in Afghanistan between March and September 2012 through a prospective, observational, research study.^22,32^ As part of ongoing efforts, these very same participants have been followed to 1-year,^22^ and now to 5-year follow-up (completed April 2017 to May 2018). In all, 212 participants were originally enrolled in combat (106 controls, 106 concussive blast).^32^ Owing to funding limitations, only 100 were invited for 5-year follow-up as was done for 1-year follow-up,^22^ and priority was placed on bringing back those who had completed prior follow-up evaluation. Two groups were enrolled, blast-related combat concussion and combat-deployed controls. Inclusion criteria for the concussion group were (1) clinical diagnosis of mild uncomplicated or concussive TBI from a blast exposure within the past 7 days made by a trained, board-certified neurologist or neurosurgeon based on the criteria from the American Congress of Rehabilitation Medicine 1993 (loss of consciousness 0-30 minutes, posttraumatic amnesia <24 hours, Glasgow coma scale 13-15, absent radiological findings), (2) injury from blast exposure within 7 days of enrollment, (3) US military, (4) ability to provide informed consent in person, (5) no contraindications to magnetic resonance imaging (MRI) such as retained metallic fragments, (6) no prior history of moderate to severe TBI based on Department of Defense criteria, (7) no prior history of mental health or psychiatric diagnosis, (8) and agreement to communicate by telephone or email and then travel to University of Washington for in-person follow-up. Inclusion criteria for the combat-deployed control group were the same except for a negative assessment for TBI and no history of blast exposure. The research protocol was approved by the institutional review board at the University of Washington and the US Army Medical Research and Material Command institutional review board. This study was conducted in accordance with the approved protocol. Written informed consent was obtained from all participants in person at each time point; no surrogate consent was allowed. See eMethods in the Supplement for further details.

For the concussion group, no intracranial abnormalities were detected on noncontrast head computed tomographic (CT) results at the time of enrollment.^32^ All concussion patients met the Department of Defense criteria for uncomplicated, mild TBI. All clinical histories were verified by study personnel taking additional clinical history and reviewing medical records. Mean (SD) time from injury to enrollment was 3.76 (1.74) days with a total range of 0 to 7 days. At the 5-year follow-up, further history was taken to assess whether there had been any additional injuries or exposures between the time of enrollment and the follow-up evaluation years later that could affect long-term outcome. Race/ethnicity was collected as a demographic variable and was identified by the participant at follow-up.

### Acute Evaluation Assessments

At the time of enrollment in Afghanistan, the following assessments were completed by both concussion and combat-deployed control participants: Rivermead Post-Concussion Symptom Questionnaire (RPCSQ),^33^ Posttraumatic Stress Disorder Check List-Military (PCL-M),^34^ Beck Depression Inventory (BDI),^35^ Combat Exposure Scale,^36^ Balance Error Scoring System (BESS),^37^ Automated Neurocognitive Assessment Metrics–Traumatic Brain Injury Military Version 4 (ANAM),^38^ and Test of Memory Malingering.^39^ Total examination time took approximately 1 hour and 15 minutes.

### 5-Year Follow-up Evaluation Assessments

In-person clinical evaluations at University of Washington included a structured neurobehavioral interview, neuropsychological battery consisting of 10 cognitive tests, and structured psychiatric evaluation with additional self-administered questionnaires. Evaluations lasted approximately 5 hours: 1 hour of standardized neurological assessment, 2 hours for cognitive testing, and 2 hours for psychiatric evaluation. Participants took all medications as prescribed by their clinicians. All tests were performed between 8 am and 5 pm in private, quiet, well-lighted rooms. All examiners were blinded to prior diagnoses and clinical histories, although during some of the interviews it may have become clear which group participants were in given endorsements of prior events. All examiners were psychometrists who underwent standardized training for administration.

Overall global disability was assessed using the Glasgow Outcome Scale Extended (GOS-E).^40,41^ Participants were instructed to consider deployment as the reference point for this interview. Poor outcome was defined as GOS-E 6 or less indicating moderate to severe disability. Additional information on the GOS-E and further neuropsychological battery details can be found in the Supplement.

The neurological assessment included a structured interview designed for patients with TBI (Neurobehavioral Rating Scale-Revised [NRS-R]^42^) scored with the 5 subdomains,^43^ 2 headache interviews capturing frequency and intensity (Migraine Disability Assessment^44^ and Headache Impact Test^45^), the Neurological Outcome Scale for TBI^46,47,48^ designed to assess focal neurological deficits associated with TBI, and a TBI history intake interview modified from the Brain Injury Screening Questionnaire,^49^ to confirm life history of head injury exposure and identify new head injuries sustained since last evaluation. Participants then completed the Quality of Life after Brain Injury^50,51^ questionnaire capturing current life satisfaction.

The psychiatric evaluation included structured interviews and self-administered questionnaires. The Clinician-Administered PTSD Scale for *Diagnostic and Statistical Manual of Mental Disorders* (Fourth Edition) (CAPS)^52^ and Montgomery-Asberg Depression Rating Scale^53^ for depression were administered as structured interviews before the participant completed the: PCL-M,^34^ BDI,^35^ Brief Symptom Inventory-Anxiety module,^54^ Insomnia Severity Index,^55^ and Michigan Alcohol Screening Test.^56^ The CAPS was scored using the rules from Blake et al.^57^

### Statistical Analysis

Data analysis was completed June to July 2018. Differences in patient characteristics between the mild blast TBI and combat-deployed control groups were assessed statistically using Mann-Whitney and Fisher exact tests as appropriate. Because many of the outcome measures had highly skewed distributions, differences in 5-year outcome measures were assessed using rank-regression modeling^58^ (ie, linear regression on the ranks of the measures) that adjusted for age, education, sex, rank, branch of service, and subsequent concussion exposure with the resulting probability values corrected for multiple comparisons^59^ within each outcome domain per Benjamini-Hochberg^60^ (2-sided *P* values <.05 were considered significant).

Univariate and multivariate predictive models in 4 domains of 5-year outcome were constructed from a predetermined set of measures collected acutely in combat using logistic regression for global disability (dichotomized GOS-E) and linear regression for neurobehavioral impairment (NRS-R), PTSD severity (CAPS), and a cumulative measure of Cognitive Performance. Overall cognitive function for each participant was defined by aggregating the 19 neuropsychological measures into a single equally weighted rank-based composite metric.^61^ This cognitive mean sample percentile was calculated by converting each score to a within-measure percentile (ranging from 0 to 100) and averaging all such percentile values within each participant. Each percentile was calculated by dividing each score's rank by the number of scores in the combined samples and multiplying by 100, after first ensuring that all measures had been transformed as necessary such that a low score corresponded to a good outcome.

A forward-stepwise selection algorithm (*P* < .05 to enter, *P* > .10 to exit) was used to establish a model for each outcome. Predictive accuracy of the logistic regression models was characterized by analyzing the receiver operating characteristic (ROC) curve and for the 3 linear regression models it was characterized by the correlation between the predicted and actual scores. In addition to the in-sample performance, bootstrap validation was used to provide a more generalizable estimate of model performance.^62^ Modeling results are reported following bootstrap validation and indicated with subscript “BV” to distinguish from initial regression results. Prediction of poor outcome was also explored if only a single acute clinical assessment could be used and assessed using ROC curve analysis, with the diagnostic threshold set at the value where the sum of the sensitivity and specificity is maximized. Five-year outcome in the global disability domain was considered poor if GOS-E was 6 or less, in the neurobehavioral domain if NRS-R was 10 or greater, and in the PTSD domain if CAPS was 65 or above. The ROC curves included both concussive blast and combat-deployed controls. When looking at prediction of 2 or more domains of poor outcome, those with poor outcome in 1 domain were excluded from the analysis.

## Results

In total, 90 of 100 invited service members completed both 0-to-7 day and 5-year evaluation: 45 concussive blast TBI and 45 combat-deployed controls (Table). Ten service members invited did not complete follow-up owing to continued deployment and related service obligations. Comparison by group of those who completed 5-year follow-up vs those who did not identified no significant differences in age, sex, branch of service, military rank, or number of deployments for the concussive blast group, whereas age and military rank slightly differed in the controls who completed follow-up (eTable 1 in the Supplement). Given the demographic differences between groups, all comparisons of clinical measures were adjusted for age, education, sex, rank, branch of service, in addition to subsequent concussion exposures followed by correction for multiple comparisons with final adjusted and corrected *P* values reported.

**Table.** Participant Characteristics at 5-Year Follow-up

Characteristic	Combat Controls (n = 45)	Concussive Blast TBI (n = 45)	*P* Value^a^
Age, mean (SD), y	34.4 (6.7)	30.6 (5.3)	.004
Education, mean (SD), y	16.6 (3.4)	13.6 (1.7)	<.001
Sex, No. (%)			
Male	33 (73)	44 (98)	.002
Female	12 (27)	1 (2)	
Race/ethnicity, No. (%)^b^			
White	35 (78)	32 (71)	.63
Black	3 (7)	2 (4)	
Hispanic/Latino	7 (16)	10 (22)	
Asian	0 (0)	1 (2)	
Branch of service, No. (%)^c^			
US Army	13 (29)	39 (87)	<.001
US Air Force	5 (11)	0 (0)	
US Marine Corps	5 (11)	5 (11)	
US Navy	22 (49)	1 (2)	
Military rank, No. (%)			
Enlisted	29 (64)	43 (96)	<.001
Officer	16 (36)	2 (4)	
No. of deployments, mean (SD)	1.5 (0.9)	1.8 (1.2)	.22
Service separation, No. (%)	16 (36)	27 (60)	.03

Abbreviation: TBI, traumatic brain injury.

^a^ Statistical significance by Mann-Whitney or Fisher exact test as appropriate.

^b^ Race/ethnicity computed as white vs other.

^c^ Branch of service computed as Army vs other.

**Figure 1.**
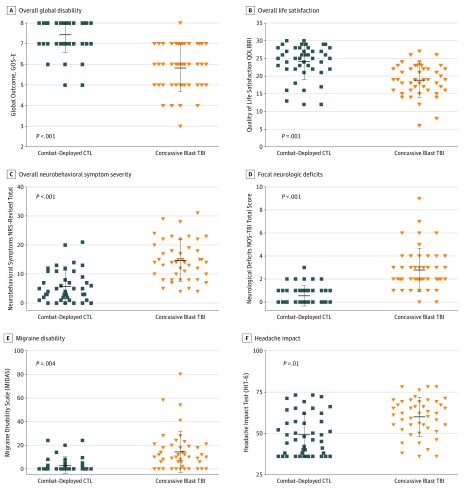
Five-Year Global Outcome, Quality of Life, and Neurobehavioral Impairment in Nonmedically Evacuated Blast Concussion and Combat-Deployed Controls (CTL)^a^ A, Overall global disability assessed by the Glasgow Outcome Scale Extended (GOS-E, 7 or 8 categorized as good outcome and GOS-E 6 or below categorized as poor outcome). B, Overall life satisfaction assessed by the Quality of Life After Traumatic Brain Injury (QOLIBRI; max, 30). C, Overall neurobehavioral symptom severity assessed by the Neurobehavioral Rating Scale Revised (NRS-R; max, 87). D, Focal neurological deficits assessed by the Neurological Outcome Scale for Traumatic Brain Injury (NOS-TBI; max, 58). Headache impairment assessed by E, the Migraine Disability Assessment (MIDAS; max, 270) and F, the Headache Impact Test (HIT-6; max, 78; min, 36). Each symbol represents an individual participant, horizontal lines indicate mean (SD) for each graph with corresponding values reported for each outcome in the legend. Lower values on panels A and B indicate worse outcome. Higher values on panels C through F indicate worse outcome. Complete summary statistics including measures of uncertainty are reported in eTable 2 in the Supplement. ^a^All *P* values adjusted.

Clinical evaluation at 5-year follow-up of service members with nonmedically evacuated concussive blast injury identified considerably worse outcomes on many measures compared with combat-deployed controls (Figure 1) (eTable 2 in the Supplement). A significantly greater number of patients with concussive blast injury presented with more severe levels of global disability (Figure 1A) (mean [SD] CTL, 7.4 [0.8]; TBI, 5.8 [1.1]; *P* < .001) and poor quality of life (Figure 1B) (mean [SD] CTL, 24.1 [4.8]; TBI, 18.9 [4.4]; *P* = .001). Overall neurobehavioral impairment (Figure 1C) (mean [SD] CTL, 5.6 [5.2]; TBI, 14.8 [6.8]; *P* < .001) was significantly elevated in patients with concussive blast injury. The frequency of focal neurological deficits was also significantly elevated in patients with concussive blast injury with the most common deficit being unilateral or bilateral hearing loss, followed by olfactory dysfunction and partial sensory loss in a lower extremity (Figure 1D) (mean [SD] CTL, 0.53 [0.76]; TBI, 2.76 [1.76]; *P* < .001). Posttraumatic headache frequency and impairment was also significantly worse in nonmedically evacuated patients with concussive blast injury (Figure 1E) (mean [SD] CTL, 3.1 [6.1]; TBI, 14.4 [16.6]; *P* = .004) (Figure 1F) (mean [SD] CTL, 49.0 [12.5]; TBI, 59.8 [11.3]; *P* = .01). Assessment for additional exposures that may have occurred between the time of enrollment to 5-year follow-up did not identify any additional TBI diagnoses; however, 18 patients with concussive blast injury and 5 combat-deployed controls reported events that would be suggestive of additional concussion although medical attention was not sought. Events were primarily fights or low speed motor vehicle/motorcycle/bicycle crashes for concussive-blast and ground level falls for both groups.

In contrast, of the 10 neuropsychological assessments administered, only the 25-foot walk was significantly different between groups (eTable 4 in the Supplement). Patients with concussive blast injury performed equivalently on all of the other cognitive test measures compared with combat-deployed controls.

**Figure 2.**
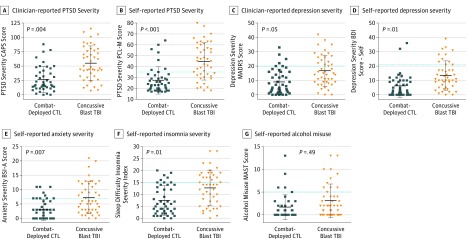
Five-Year Psychiatric Symptom Severity in Nonmedically Evacuated Blast Concussion and Combat-Deployed Controls (CTL)^a^ TBI Indicates traumatic brain injury. A, Posttraumatic stress disorder (PTSD) severity assessed by the clinician-administered PTSD scale for DSM IV (CAPS; max, 136), and B, the self-administered PTSD checklist military version (PCL-M; max, 85; Min, 17). C, Depression severity assessed by the Montgomery-Asberg depression rating scale (MADRS; max, 60), and D, the self-administered Beck Depression Inventory (BDI; max, 63). E, Anxiety symptom severity assessed by the Brief Symptom Inventory Anxiety module (BSI-A; max, 24). F, Severity of poor sleep assessed by the Insomnia Severity Index (ISI; max, 28). G, Alcohol misuse assessed by the Michigan Alcohol Screening Test (MAST; max, 22). Dotted lines indicate the threshold for moderate to severe symptomatology for each evaluation. Each symbol represents an individual participant, horizontal lines indicate mean (SD) for each graph with corresponding values reported for each outcome in the legend. Higher values on each measure indicate worse outcome. Complete summary statistics including measures of uncertainty are reported in eTable 3 in the Supplement. ^a^All *P* values adjusted.

Examination on a variety of psychiatric measures did reveal significant long-term symptomatology in nonmedically evacuated patients with concussive blast injury related to the blast exposure compared with combat-deployed controls (Figure 2) (eTable 3 in the Supplement). There was significant PTSD symptom severity identified by both clinical interview (CAPS) and self-administered questionnaire (PCL-M) (Figure 2A) ( mean [SD] CTL, 26.8 [21.7]; TBI, 55.0 [29.1]; *P* = .004) (Figure 2B) (mean [SD] CTL, 27.4 [11.8]; TBI, 44.7 [16.6]; *P* < .001). Depressive symptoms were also significantly elevated on the clinical interview (Montgomery-Asberg Depression Rating Scale) and questionnaire (BDI) but to a lesser extent than PTSD symptoms (Figure 2C) (mean [SD] CTL, 9.0 [8.5]; TBI, 16.7 [10.6]; *P* = .05) (Figure 2D) (mean [SD] CTL, 6.1 [7.7]; TBI, 13.4 [9.8]; *P* = .01). Self-endorsed anxiety symptoms (Brief Symptom Inventory-Anxiety Module) and sleep impairment were significantly worse, whereas there was no difference in alcohol misuse between the 2 groups (Figure 2E) (mean [SD] CTL, 3.0 [3.5]; TBI, 7.2 [5.4]; *P* = .007) (Figure 2F) (mean [SD] CTL, 7.4 [6.0]; TBI, 12.7 [7.2]; *P* = .01) (Figure 2G) (mean [SD] CTL, 1.7 [2.6]; TBI, 3.1 [3.6]; *P* = .49). Importantly, 35 patients (78%) with concussive blast injury and 25 combat-deployed controls (56%) reported seeking mental health services but only 13 patients (28%) with concussive blast injury and 18 combat-deployed controls (40%) reported sustained symptom resolution.

**Figure 3.**
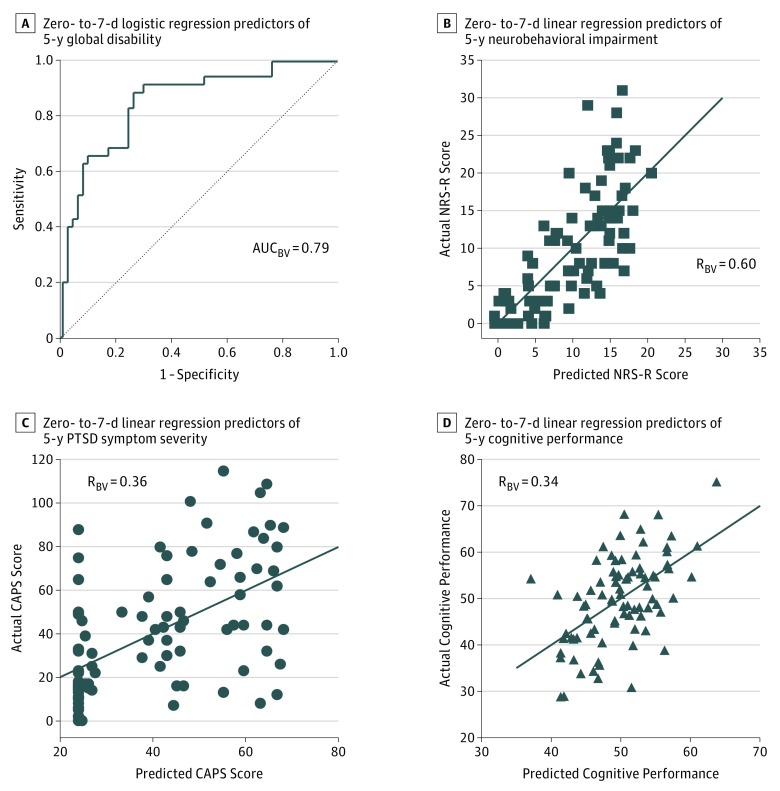
Acute Predictors of 5-Year Outcome for Global Disability, Neurobehavioral Impairment, Posttraumatic Stress Disorder (PTSD) Symptom Severity, and Cognitive Performance AUC_BV_ Indicates area under the curve following bootstrap validation; BDI, Beck Depression Inventory; CAPS, Counseling and Psychological Services PTSD scale; NRS-R, Neurobehavioral Rating Scale-Revised; R_BV_, correlation following bootstrap validation. A, Logistic regression acute predictors of 5-year global disability (AUC_BV_ = 0.79; model parameters: TBI diagnosis, OR = 7.86; *P* = .001; Concussion Symptoms [Rivermead Post-Concussion Symptom Questionnaire; RPCSQ], OR = 1.06; *P* = .008). B, Linear regression acute predictors of 5-year neurobehavioral impairment (R_BV_ = 0.60; model parameters: TBI diagnosis, B = 7.01; *P* < .001; enlisted status, B = −4.60; *P* = .004; depression symptoms [BDI], B = 0.25; *P* = .006; processing speed [Automated Neuropsychological Assessment Metrics; ANAM-PRT], B = 0.10; *P* = .02; visuospatial memory [Automated Neuropsychological Assessment Metrics Matching to Sample; ANAM-MTS], B = −0.19; *P* = .001). C, Linear regression acute predictors of 5-year PTSD symptom severity (R_BV_ = 0.36; model parameters: TBI diagnosis, B = 17.59; *P* = .006; concussion symptoms [RPCSQ], B = 0.72; *P* = .005). D, Linear regression acute predictors of 5-year cognitive performance (R_BV_ = 0.34; model parameters: TBI diagnosis, B = 5.35; *P* = .007; working memory [ANAM-MTP], B = −0.45; *P* = .001; balance performance [Balance Error Scoring System], B = −0.27; *P* = .02). See eTables 5 to 8 in the Supplement for complete details by parameter for all univariate and multivariate models including measures of uncertainty. Dotted line panel A represents unity line and graph lines in panels B to D represent regression line for each model.

Owing to the number of nonmedically evacuated patients with concussive blast injury with poor 5-year outcomes, we investigated if any of the acutely collected measures could predict these outcomes. Four primary outcome domains were examined using univariate and then forward-stepwise selection multivariate modeling for global disability, neurobehavioral impairment, PTSD symptom severity, and overall cognitive function (Figure 3) (eTables 5-8 in the Supplement). Traumatic brain injury diagnosis (odds ratio [OR], 7.86; *P* = .001) and concussion symptoms (RPCSQ; OR, 1.06; *P* = .008) collected acutely in theater best predicted 5-year global disability (AUC_BV_ = 0.79 indicating very strong predictive strength) (Figure 3A) (eTable 5 in the Supplement). Traumatic brain injury diagnosis (B = 7.01, *P* < .001), being enlisted (B = 4.06, *P* = .004), depression symptoms (BDI, B = 0.25, *P* = .006), and information processing speed (ANAM-PRT, B = 0.10, *P* = .02), and visual spatial memory (ANAM-MTS, B = −0.19, *P* = .001) collected acutely in theater best predicted 5-year neurobehavior impairment (R_BV_ = 0.60 indicating strong predictive strength) (Figure 3B) (eTable 6 in the Supplement). Traumatic brain injury diagnosis (B = 17.59, *P* = .006) and concussion symptoms (RPCSQ, B = 0.72, *P* = .004) collected acutely best predicted 5-year PTSD severity (R_BV_ = 0.36 indicating moderate predictive strength) (Figure 3C) (eTable 7 in the Supplement). Traumatic brain injury diagnosis (B = 5.35, *P* = .007), working memory (ANAM-MTP, B = −0.45, *P* = .001), and balance performance (BESS, B = −0.27, *P* = .02) best predicted 5-year cognitive function (R_BV_ = 0.34 indicating moderate prediction strength) (Figure 3D) (eTable 8 in the Supplement).

**Figure 4.**
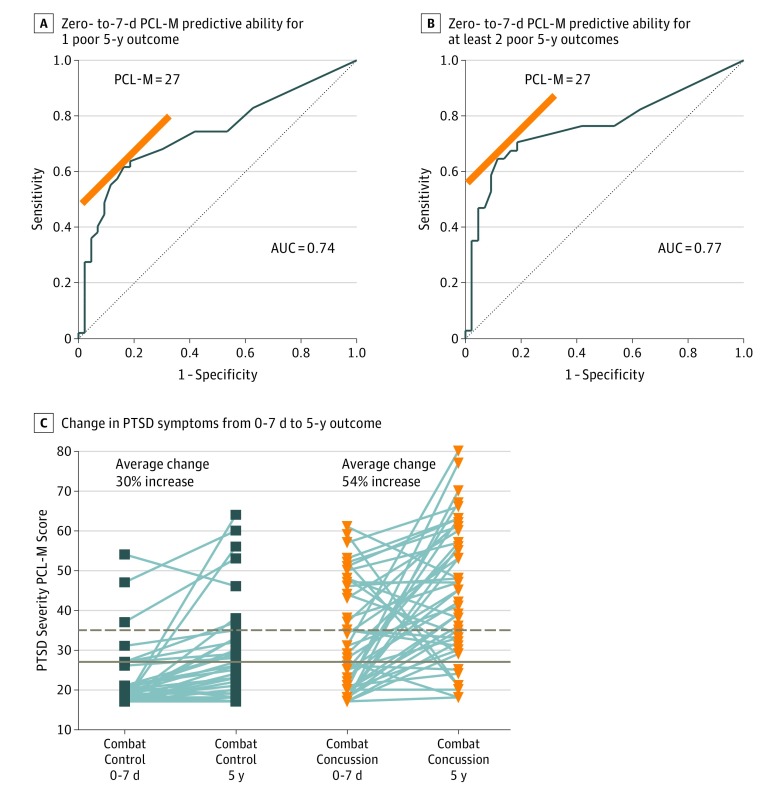
Acute PCL-M Score Threshold for Prediction of 5-Year Multidomain Poor Outcome If only a single evaluation tool could be used in combat, the Posttraumatic Stress Disorder (PTSD) Checklist Military Version (PCL-M) was found to have the best predictive ability to inform poor global disability, neurobehavioral impairment, and PTSD outcome. A threshold of 27 provided the best cut point regardless of whether the logistic regression model sought to identify only A, 1 domain of poor outcome (area under the curve [AUC] = 0.74) or B, multiple domains (AUC = 0.77), with both indicating fair predictive strength. C. Self-administered PTSD Checklist Military Version was completed at 0 to 7 days postinjury in combat and at 5-year outcome for direct comparison over time (PCL-M; max, 85). Line plots indicate change in score for each concussive blast patient and combat-deployed control participant. The brown dashed line indicates the current clinical threshold whereas the solid brown line indicates the threshold identified by the current prediction modeling. See eTable 9 in the Supplement for complete details of model optimization parameters for this multidomain outcome prediction including measures of uncertainty.

Given the austere nature of the acute critical care environment in combat, we next asked the question, if only a single evaluation tool could be used following concussion diagnosis, what measure would best predict these domains of 5-year outcome. Acute collection of PTSD symptoms (PCL-M) provided the strongest predictive ability for the 3 domains where patients with concussive blast injury had worse impairment (global disability, AUC = 0.68; neurobehavioral impairment, R = 0.68; PTSD symptoms, R = 0.58; eTable 9A-C in the Supplement). Investigation of a PCL-M threshold for this predictive model identified a score of 27 as the optimal cut point in predicting 1 poor outcome (AUC = 0.74) (Figure 4A) or more than 1 poor outcome (AUC = 0.77) (Figure 4B) indicating fair predictive strength. Examination of the PCL-M scores in the very same participants for each group collected acutely in combat and 5 years later revealed an overall average increase of 30% in combat-deployed controls and 54% in nonmedically evacuated patients with concussive blast injury with most patients with concussive blast injury noting increased symptom burden (Figure 4C). Percent change was derived by taking the difference between the 2 scores and dividing by the initial score.

## Discussion

At 5-year follow-up, service members with nonmedically evacuated concussive blast injury fared significantly more poorly than combat-deployed controls on measures of global disability, neurobehavioral impairment, and psychiatric symptom severity whereas cognitive test performance was similar. Predictors of overall 5-year clinical outcome across 4 different domains primarily encompassed TBI diagnosis and acute symptoms of mental health and concussion, not acute cognitive performance, age, sex, number of prior deployments, or number of subsequent concussions. When considering only a single assessment tool, the PCL-M for PTSD symptoms collected acutely in theater best predicted domains of outcome in combination with concussion diagnosis at a cut point of 27 that is lower than the threshold typically used to indicate clinical significance of 35.^34^ Both combat-deployed controls and, to a greater extent, nonmedically evacuated patients with concussive blast injury had a substantial increase in PTSD symptom severity over the 5-year period suggesting an evolution, not resolution, of mental health burden.

These results are comparable to our previous 5-year findings in patients with medically evacuated concussive blast injury^31^ suggesting a more universal effect of these concussive blast exposures irrespective of evacuation status. Like the medically evacuated cohort, this group of service members with nonmedically evacuated concussion was found to have a much greater level of global disability than previously reported in prospective studies of comparable civilian patients with mild TBI, even those with multisystem trauma.^63,64^ This has important implications for the translatability of these combined concussive blast findings because there are far greater numbers of service members who sustain concussion exposures in combat that are not medically evacuated. This has been a limitation of our prior work and here we provide prospective, longitudinal evidence for the progression in this nonmedically evacuated blast concussion population. Furthermore, the disproportionate number of patients with concussive blast injury with subsequent exposures is in line with prior work reporting that brain injury is in fact a risk factor for further TBI.^65,66^

Strengths of this study include the prospective, observational, longitudinal study design, evaluation in multiple domains of function at both time points, enrollment of a combat-deployed control group for comparison with the patients with concussive blast injury, concussion diagnosis made by trained clinicians in combat not based on self-report recollection, collection of medical history accounting for subsequent exposures that may have occurred between points of evaluation, and clinical evaluations made by blinded examiners.

### Limitations

Limitations of the study include the modest sample size, lack of demographic matching, heterogeneous treatment centers in which these patients have sought care in the United States, and unmeasured covariates that may influence these findings such as preinjury characteristics including military operation specialty, or postinjury behavioral health referrals, use of sick leave, or unmeasured alcohol use not identified on the alcohol screening test completed as part of the study. In addition, because of the sample size, it is possible that type II error may have contributed to the neuropsychological results in which few differences between groups were observed. Furthermore, the study was designed to explore predictors of long-term outcome without necessarily causal relation, so the predictors identified should not be assumed to be causally related to the concussion exposure. It should also be noted that while the predictive models were assessed for model performance by bootstrap validation, we cannot rule out the possibility of unappreciated overfitting and therefore replication in an independent cohort should be completed before the models are considered fully validated.

## Conclusions

The results support a more focused and efficient acute screening of mental health in theater following TBI diagnosis as strong indicators of poor long-term outcome. The PCL-M was found to be the most informative measure in predicting long-term functional outcome following blast-related mild TBI and can be completed within 1 to 2 minutes, supporting its utility in an acute triage environment. Future studies should examine whether early intervention informed by this acute assessment may prevent some of the adverse long-term outcomes associated with blast-related mild TBI.

## Notes

Supplement. **eMethods** **eTable 1.** Comparison of Participant Characteristics at Enrollment, Follow Up vs. No Follow Up **eTable 2.** Summary Statistics for Figure 1. 5-Year Global Outcome, Quality of Life and Neurobehavioral Impairment in Non-Medically Evacuated Blast Concussion and Combat-deployed Controls **eTable 3.** Summary Statistics for Figure 2. 5-Year Psychiatric symptom severity in Non-Medically Evacuated Blast Concussion and Combat-deployed Controls **eTable 4.** Neuropsychological Test Performance at 5-Year Follow Up **eTable 5.** Logistic Regression, Prediction of 5-Year Poor Global Outcome **eTable 6.** Linear Regression Prediction of 5-Year Neurobehavioral Impairment **eTable 7.** Linear Regression Prediction of 5-Year PTSD Symptom Severity **eTable 8.** Linear Regression Prediction of 5-Year Cognitive Performance **eTable 9.** Prediction Modeling Optimization **eReferences**
