# Supplementary material for: Association Between 5-Year Clinical Outcome in Patients With Nonmedically Evacuated Mild Blast Traumatic Brain Injury and Clinical Measures Collected Within 7 Days Postinjury in Combat
Source: JAMA Netw Open. 2019 Jan 4;2(1):e186676. doi: 10.1001/jamanetworkopen.2018.6676 (PMC6324322; doi:10.1001/jamanetworkopen.2018.6676)
Supplement: Supplement. — eMethods eTable 1. Comparison of Participant Characteristics at Enrollment, Follow Up vs. No Follow Up eTable 2. Summary Statistics for Figure 1. 5-Year Global Outcome, Quality of Life and Neurobehavioral Impairment in Non-Medically Evacuated Blast Concussion and Combat-deployed Controls eTable 3. Summary Statistics for Figure 2. 5-Year Psychiatric symptom severity in Non-Medically Evacuated Blast Concussion and Combat-deployed Controls eTable 4. Neuropsychological Test Performance at 5-Year Follow Up eTable 5. Logistic Regression, Prediction of 5-Year Poor Global Outcome eTable 6. Linear Regression Prediction of 5-Year Neurobehavioral Impairment eTable 7. Linear Regression Prediction of 5-Year PTSD Symptom Severity eTable 8. Linear Regression Prediction of 5-Year Cognitive Performance eTable 9. Prediction Modeling Optimization eReferences [file jamanetwopen-2-e186676-s001.pdf]

Supplementary Online Content

Mac Donald CL, Barber J, Patterson J, et al. Association between 5-year clinical outcome in patients with nonmedically evacuated mild blast traumatic brain injury and clinical measures collected within 7 days postinjury in combat. *JAMA Netw Open*. 2019;2(1):e186676. doi:10.1001/jamanetworkopen.2018.6676

eMethods

eTable 1. Comparison of Participant Characteristics at Enrollment, Follow Up vs. No Follow Up

eTable 2. Summary Statistics for Figure 1. 5-Year Global Outcome, Quality of Life and Neurobehavioral Impairment in Non-Medically Evacuated Blast Concussion and Combat-deployed Controls

eTable 3. Summary Statistics for Figure 2. 5-Year Psychiatric symptom severity in Non-Medically Evacuated Blast Concussion and Combat-deployed Controls

eTable 4. Neuropsychological Test Performance at 5-Year Follow Up

eTable 5. Logistic Regression, Prediction of 5-Year Poor Global Outcome

eTable 6. Linear Regression Prediction of 5-Year Neurobehavioral Impairment

eTable 7. Linear Regression Prediction of 5-Year PTSD Symptom Severity

eTable 8. Linear Regression Prediction of 5-Year Cognitive Performance

eTable 9. Prediction Modeling Optimization

eReferences

This supplementary material has been provided by the authors to give readers additional information about their work.

© 2019 Mac Donald CL et al. *JAMA Netw Open.*

1. eMETHODS

1.1 Participant Compensation

Active duty military subjects were not paid for follow up participation, though travel expenses to University of Washington in Seattle were covered. Subjects who had subsequently separated from the service at the time of follow-up were paid $250 for participation in addition to having their travel expenses covered.

1.2 Safety and Data Monitoring

Subjects were assigned a random 5-digit code number to protect confidentiality and all research data was identified by code number only. A board-certified psychiatrist (J. Fann) was immediately available in case an evaluation exacerbated mental health symptoms. No exacerbations requiring medical intervention occurred, though additional support from study staff was required on several occasions.

For clinical evaluations, the principal investigator audited 1 in 10 randomly selected subjects' data sets to ensure that data was scored and entered correctly. These audits revealed only minor discrepancies in scoring criteria which were then corrected across the entire cohort of subjects.

1.3 Acute Evaluation Assessments

The severity of post-concussive symptoms was measured by the Rivermead Post-Concussion Symptom Questionnaire (RPCSQ)[1], a self-administered questionnaire assessing 16 post-concussive symptoms on a scale of 0 (none) to 4 (severe) covering three domains: cognitive (memory and concentration difficulties), emotional (anxiety, restlessness and depression) and somatic (fatigue, headache, dizziness, nausea, sleep disturbance and changes in vision). Symptoms of post-traumatic stress disorder (PTSD), anxiety and mood changes were assessed using the Post-traumatic Stress Disorder Check List-Military (PCL-M)[2] and Beck Depression Inventory (BDI)[3]. The PCL-M is a 17 item self-administered questionnaire tying symptom ratings to events experienced during military service, using a scale of 1 (not at all) to 5 (extremely). The BDI is a self-administered 21 item questionnaire corresponding to symptoms of depression rated on a severity scale of 0 (no symptoms) to 3 (severe symptoms). Reports of wartime stressors experienced by combatants were measured using the Combat Exposure Scale (CES)[4], a 7-item scale with 5-response points (1 is "no", 2 is "1 to 3 times", 3 is "4 to 12 times", 4 is "13 to 50 times", and 5 is "51+ times"), each item being weighted differently based on the severity of the experience, the total scores ranging from 0−41. Severity of balance impairment was tested using the Balance Error Scoring System (BESS)[5]. The BESS is a clinician administered balance test which includes single, double and tandem stance assessment on firm and foam (unstable) surfaces, each held for 20 seconds, with the participant's hands on the hips and eyes closed. The final score is a representation of cumulative errors. The Automated Neurocognitive Assessment Metrics – Traumatic Brain Injury Military Version 4 (ANAM)[6] is sanctioned by the Department of Defense for baseline neurocognitive assessment in all deploying troops and it is also available in the deployed setting. The ANAM includes a collection of cognitive modules including simple reaction time (SRT) and repeat simple reaction time (SRTR) for basic neural processing, Code substitution learning (CSL) for associative learning, procedural reaction time (PRT) for processing speed, mathematical processing (MTP) for working memory, matching to sample (MTS) for visual spatial memory and code substitution delayed (CSD) for delayed memory. The cognitive modules are preceded by sleepiness and mood scales. Level of examination effort was measured using the Test of Memory Malingering (TOMM)[7], which is a clinician administered tool designed to assist in determining effort[7]. The testing paradigm involved a single TOMM trial for subjects with a score higher or equal to 45 and a second trial for subjects with a first TOMM score lower than 45. Subjects with TOMM score lower than 45 on both consecutive TOMM trials were excluded from analysis for possible poor effort during testing.

1.4 Glasgow Outcome Scale Extended

The GOS-E is scored from 1-8: 1=dead, 2=vegetative, 3-4=severe disability, 5-6=moderate disability, 7-8=good recovery. Moderate disability (GOS-E = 5-6) is defined as one or more of the following: 1) inability to work to previous capacity 2) inability to resume much of regular social and leisure activities outside the home 3) psychological problems which have frequently resulted in ongoing family disruption or disruption of friendships. Severe disability (GOS-E = 3-4) is defined as one or more of the following: 1) inability to drive and/or travel locally without assistance 2) inability to shop or run errands without assistance 3) support required for activities of daily living. Standardized, structured interviews were performed per published guidelines.[8] Participants were instructed to consider deployment as the reference point for this interview.

© 2019 Mac Donald CL et al. *JAMA Netw Open.*

1.5 Neuropsychological Test Battery

The neuropsychological test battery consisted of the following: Conner's Continuous Performance Test II [9], a computer-based assessment of attention, impulsivity, reaction time, and vigilance; the California Verbal Learning Test II [10], an assessment of verbal declarative memory; the 25 hole grooved pegboard test [11], an assessment of upper extremity motor speed and coordination; a timed 25 foot walk, an assessment for motor strength, balance, and coordination; the Trail Making test [12], an assessment of visual scanning and mental flexibility; the Controlled Oral Word Association test [13], an assessment of verbal fluency; the Wechsler Test of Adult Reading [14] as an estimate of pre-injury verbal intelligence; the Iowa Gambling Test[15], a computer-based assessment of impulsivity and decision making; the D-KEFS Color-Word Interference Test [16], a multi-domain assessment of executive function similar to the Stroop test; and the Ruff-Light Trail Learning Test[17], an assessment of visual-spatial memory. A relatively easy forced choice test embedded in the California Verbal Learning Test was used to assess adequacy of effort.

© 2019 Mac Donald CL et al. *JAMA Netw Open*.

eTable 1. Comparison of Participant Characteristics at Enrollment, Follow Up vs. No Follow Up						
Characteristic	Combat CTL			Concussive Blast TBI		
	Followed (N=45)	Not-Followed (N=60)	P-Value	Followed (N=45)	Not-Followed (N=62)	P-Value
**Age in years:**						
mean (stdev)	29.7 (6.7)	26.4 (4.1)	0.02 MWU	26.0 (5.4)	25.1 (4.4)	0.61 MWU
**Gender - no (%)**						
Male	33 (73%)	48 (80%)	0.49 Fisher's	44 (98%)	61 (98%)	1.00 Fisher's
Female	12 (27%)	12 (20%)		1 (2%)	1 (2%)	
**Branch of Service - no (%)**						
US Army	13 (29%)	27 (45%)	0.11 Fisher's	39 (87%)	49 (80%)	0.44 Fisher's
US Air Force	5 (11%)	7 (12%)		0 (0%)	0 (0%)	
US Marine Corps	5 (11%)	6 (10%)		5 (11%)	12 (20%)	
US Navy	22 (49%)	20 (33%)		1 (2%)	0 (0%)	
**Military Rank - no (%)**						
Enlisted	29 (64%)	52 (87%)	0.01 Fisher's	43 (96%)	57 (93%)	1.00 Fisher's
Officer	16 (36%)	8 (13%)		2 (4%)	4 (7%)	
**Number of Deployments**						
mean (stdev)	1.8 (1.2)	1.8 (1.2)	0.86 MWU	2.0 (1.8)	2.2 (1.5)	0.16 MWU

Statistical significance by Mann-Whitney or Fisher's exact test as appropriate.
Branch of service computed as Army vs. Other.

© 2019 Mac Donald CL et al. *JAMA Netw Open*.

eTable 2. Summary Statistics for Figure 1. 5-Year Global Outcome, Quality of Life and Neurobehavioral Impairment in Non-Medically Evacuated Blast Concussion and Combat-deployed Controls						
**Figure 1 Panel Outcome Measure**	**Combat CTL (n=45)** Mean ± Std. Dev.	**Concussive Blast (n=45)** Mean ± Std. Dev.	**Adjusted P-value [1]**	**Adjusted and Corrected P-value [2]**	**Adjusted Difference [3]**	**95% CI for Adjusted Difference**
A. Global Outcome: GOS-E	7.44 ± 0.81	5.84 ± 1.11	<.001	<.001	-1.39	(-1.95, -0.83)
B. Quality of Life Satisfaction: QOLIBRI	24.07 ± 4.76	18.87 ± 4.43	<.001	.001	-4.55	(-7.21, -1.89)
C. Neurobehavioral Symptoms: NRS-R	5.62 ± 5.21	14.76 ± 6.81	<.001	<.001	8.19	(4.72, 11.66)
D. Neurological Deficits: NOS-TBI Score	0.53 ± 0.76	2.76 ± 1.76	<.001	<.001	1.76	(0.96, 2.56)
E. Migraine Disability Scale (MIDAS)	3.12 ± 6.09	14.4 ± 16.6	.002	.004	10.65	(3.19, 18.11)
F. Headache Impact Test (HIT-6)	49.0 ± 12.5	59.8 ± 11.3	.008	.01	9.71	(2.88, 16.53)

1. Rank-regression significance is adjusted for age, education, gender, rank, branch of service, number of subsequent concussion exposures
2. Significance is further corrected for multiple comparisons (Benjamini-Hochberg, m=6)
3. Adjusted difference is a parametric regression estimate, adjusted for age, education, gender, rank, branch of service, and subsequent exposure (but not multiple comparisons)

© 2019 Mac Donald CL et al. *JAMA Netw Open*.

eTable 3. Summary Statistics for Figure 2. 5-Year Psychiatric symptom severity in Non-Medically Evacuated Blast Concussion and Combat-deployed Controls						
**Figure 2 Panel Outcome Measure**	**Combat CTL (n=45)** Mean ± Std. Dev.	**Concussive Blast (n=45)** Mean ± Std. Dev.	**Adjusted P-value [1]**	**Adjusted and Corrected P-value [2]**	**Adjusted Difference [3]**	**95% CI for Adjusted Difference**
A. PTSD Severity: CAPS Score	26.8 ± 21.7	55.0 ± 29.1	.002	.004	24.56	(9.61, 39.51)
B. PTSD Severity: PCL-M Score	27.4 ± 11.8	44.7 ± 16.6	<.001	<.001	17.03	(8.53, 25.53)
C. Depression Severity: MADRS Score	9.02 ± 8.50	16.7 ± 10.6	.04	.05	5.74	(0.13, 11.35)
D. Depression Severity: BDI Score	6.13 ± 7.65	13.42 ± 9.76	.01	.01	5.08	(0.09, 10.08)
E. Anxiety Severity: BSI-A Score	3.00 ± 3.50	7.22 ± 5.44	.005	.007	4.07	(1.37, 6.77)
F. Sleep Difficulty: Insomnia Severity	7.44 ± 5.95	12.69 ± 7.16	.01	.01	5.30	(1.44, 9.16)
G. Alcohol Misuse: MAST Score	1.69 ± 2.58	3.09 ± 3.58	.49	.49	1.22	(-0.59, 3.04)

1. Rank-regression significance is adjusted for age, education, gender, rank, branch of service, number of subsequent concussion exposures
2. Significance is further corrected for multiple comparisons (Benjamini-Hochberg, m=7)
3. Adjusted difference is a parametric regression estimate, adjusted for age, education, gender, rank, branch of service, and subsequent exposure (but not multiple comparisons)

© 2019 Mac Donald CL et al. *JAMA Netw Open*.

eTable 4. Neuropsychological Test Performance at 5-Year Follow Up						
**Assessment**	**Combat CTL (n=45)**	**Concussive Blast TBI (n=45)**	**Adjusted P-value [1]**	**Adjusted and Corrected P-value [2]**	**Adjusted Difference [3]**	**95% CI for Adjusted Difference**
25-Foot Walk (seconds): *Motor Strength, Balance, Coordination*	4.1 ± 0.5	4.6 ± 0.9	.001	.02	0.67	(0.25, 1.09)
Conners' Continuous Performance Test II						
Omission Errors (T-score): *Attention Lapses*	47.0 ± 6.7	47.8 ± 6.0	.22	.52	-1.33	(-5.03, 2.38)
Commission Errors (T-score): *Impulsivity*	49.3 ± 10.1	51.4 ± 8.5	.69	.88	0.64	(-4.92, 6.21)
Hit Rate (T-score): *Reaction Time*	51.6 ± 7.0	51.9 ± 6.9	.62	.85	0.40	(-3.77, 4.56)
Hit Rate Block Change (T-score): *Sustained Vigilance*	51.1 ± 8.9	54.3 ± 8.2	.54	.85	1.28	(-3.87, 6.43)
Wechsler Test of Adult Reading (Standard Score)						
(Estimate of Pre-injury Verbal Intelligence)	108 ± 11	102 ± 14	.63	.85	-2.63	(-9.77, 4.51)
California Verbal Learning Test II						
Long-Delay Free Recall (Standard Score): *Verbal Memory*	0.24 ± 0.93	-0.27 ± 1.09	.10	.48	-0.53	(-1.15, 0.08)
Total Intrusions (Standard Score): *Falsely Recalled Items*	-0.11 ± 0.91	-0.18 ± 0.85	.48	.84	0.17	(-0.34, 0.69)
List B vs. List A (Z-score): *Proactive Memory Interference*	-0.08 ± 1.22	-0.08 ± 0.83	.49	.84	0.21	(-0.41, 0.84)
Ruff Light Trail Learning Test						
Total Trials Correct (T-score): *Visuospatial Learning*	51.5 ± 9.9	49.6 ± 9.5	.08	.48	-2.95	(-8.76, 2.86)
Long Delay Trial Correct: *Visuospatial Memory*	14.4 ± 1.1	14.5 ± 1.0	.92	.94	0.04	(-0.57, 0.65)
Grooved Pegboard (Motor Speed & Coordination)						
Average Dom & Non-Dom Time (seconds)	64.8 ± 10.2	72.3 ± 11.9	.16	.51	4.52	(-2.11, 11.15)
Trail Making Test						
Trails A time (seconds): *Visual Scanning, Coordination*	21.4 ± 4.9	27.6 ± 11.7	.010	.09	8.36	(3.11, 13.62)
Trails B time (seconds): *Mental Flexibility*	55.0 ± 16.8	63.1 ± 19.3	.17	.51	7.47	(-3.48, 18.42)
Controlled Oral Word Association Total Score: *Verbal Fluency*	47.0 ± 11.1	47.6 ± 11.6	.82	.94	1.00	(-5.82, 7.82)
DKEFS Color Word Interference: *Executive Function*						
Trial 1 + Trial 2 (scaled score): *Naming, Reading*	20.8 ± 5.1	19.4 ± 4.5	.94	.94	0.43	(-2.37, 3.23)
Trial 3 (scaled score): *Inhibition*	10.8 ± 2.5	10.8 ± 7.4	.36	.76	0.24	(-3.03, 3.51)
Trial 4 (scaled score): *Inhibition Switching*	10.4 ± 2.8	9.0 ± 2.5	.19	.51	-0.79	(-2.33, 0.75)
Iowa Gambling Task Net Trials (T-score): *Monetary decision making*	51.0 ± 9.6	50.7 ± 11.5	.91	.94	-0.04	(-6.42, 6.34)

1. Rank-regression significance is adjusted for age, education, gender, rank, branch of service, number of subsequent concussion exposures
2. Significance is further corrected for multiple comparisons (Benjamini-Hochberg, m=19)
3. Adjusted difference is a parametric regression estimate, adjusted for age, education, gender, rank, branch of service, and subsequent exposure (but not multiple comparisons)

© 2019 Mac Donald CL et al. *JAMA Netw Open*.

eTable 5. Logistic Regression<br>Prediction of 5-Year Poor Global Outcome (GOSE 6 or Less vs. GOSE 7-8)													
**0-7 Day Predictors**		**Univariate**						**Multivariate AUC = 0.85, AUC$_{BV}$ = 0.79**					
		**B**	**SE(B)**	**OR**	**p-value**	**95% CI for OR**		**B**	**SE(B)**	**OR**	**p-value**	**95% CI for OR**	
**Demographics & 0-7 Day Outcomes**	Age in Combat	-0.09	0.04	0.92	.03	0.85	0.99				.46		
	Officer vs. Enlisted	-2.72	1.06	0.07	.01	0.01	0.52				.13		
	Female vs. Male	-1.42	0.80	0.24	.08	0.05	1.17				.59		
	Branch of Service	1.91	0.53	6.72	<.001	2.40	18.83				.68		
	TBI Diagnosis	2.77	0.57	16.00	<.001	5.23	48.91	2.06	0.62	7.86	.001	2.32	26.57
	Number of Deployments	-0.01	0.14	0.99	.96	0.75	1.31				.48		
	Number of Subsequent Concussions	0.70	0.28	2.01	.01	1.15	3.50				.13		
	Concussion Symptoms (RPCSQ)	0.10	0.02	1.10	<.001	1.06	1.15	0.06	0.02	1.06	.008	1.02	1.12
	PTSD Symptom Severity (PCL-M)	0.09	0.02	1.09	<.001	1.05	1.14				.52		
	Depression Symptom Severity (BDI)	0.12	0.04	1.13	.001	1.05	1.21				.95		
	Combat Intensity (CES)	0.09	0.02	1.09	<.001	1.04	1.14				.96		
	Postural Stability (BESS)	0.01	0.03	1.01	.77	0.95	1.07				.32		
**0-7 Day ANAM Testing**	Sleep Impairment	0.66	0.19	1.93	.001	1.33	2.80				.98		
	Simple Reaction Time	0.01	0.00	1.01	.07	1.00	1.01				.88		
	Simple Reaction Time -R	0.00	0.00	1.00	.50	1.00	1.00				.93		
	Processing Speed (PRT)	-0.02	0.01	0.98	.15	0.96	1.01				.19		
	Associative Learning (CSL)	-0.03	0.02	0.97	.09	0.94	1.00				.94		
	Delayed Memory (CSD)	-0.03	0.02	0.97	.11	0.95	1.01				.76		
	Working Memory (MTP)	-0.08	0.04	0.92	.02	0.86	0.99				.86		
	Visuospatial Memory (MTS)	-0.04	0.02	0.96	.05	0.92	1.00				.15		

B = Coefficient from the logistic regression; SE(B) = Standard error of B; OR = Odds ratio; CI = Confidence Interval; AUC = Area under the ROC curve; AUC$_{BV}$ = Area under the ROC curve from bootstrap validation.
Multivariate models selected using a stepwise algorithm (p<.05 to enter, >.10 to exit).
Education was not always available in theatre so officer vs. enlisted was used as a surrogate.

© 2019 Mac Donald CL et al. *JAMA Netw Open*.

eTable 6. Linear Regression
Prediction of 5-Year Neurobehavioral Impairment

0-7 Day Predictors		Univariate					Multivariate R = 0.73, R_{BV} = 0.60				
		B	SE(B)	p-value	95% CI for B		B	SE(B)	p-value	95% CI for B	
Demographics & 0-7 Day Outcomes	Age in Combat	-0.32	0.12	.01	-0.56	-0.08			.96		
	Officer vs. Enlisted	-8.29	1.80	<.001	-11.88	-4.71	-4.60	1.54	.004	-7.67	-1.54
	Female vs. Male	-5.53	2.21	.01	-9.92	-1.14			.84		
	Branch of Service	5.20	1.53	.001	2.16	8.24			.73		
	TBI Diagnosis	9.13	1.28	<.001	6.59	11.68	7.01	1.32	<.001	4.39	9.63
	Number of Deployments	0.49	0.53	.35	-0.55	1.54			.70		
	Number of Subsequent Concussions	2.09	0.86	.02	0.39	3.80			.52		
	Concussion Symptoms (RPCSQ)	0.29	0.05	<.001	0.19	0.40			.28		
	PTSD Symptom Severity (PCL-M)	0.27	0.06	<.001	0.16	0.39			.40		
	Depression Symptom Severity (BDI)	0.41	0.11	<.001	0.20	0.62	0.25	0.09	.006	0.07	0.42
	Combat Intensity (CES)	0.34	0.07	<.001	0.20	0.47			.50		
	Postural Stability (BESS)	0.05	0.10	.65	-0.16	0.26			.39		
0-7 Day ANAM Testing	Sleep Impairment	2.16	0.59	<.001	0.99	3.34			.72		
	Simple Reaction Time	0.01	0.01	.27	-0.01	0.02			.24		
	Simple Reaction Time -R	0.01	0.01	.27	0.00	0.02			.89		
	Processing Speed (PRT)	-0.07	0.05	.13	-0.17	0.02	0.10	0.04	.02	0.02	0.18
	Associative Learning (CSL)	-0.06	0.06	.29	-0.18	0.06			.75		
	Delayed Memory (CSD)	-0.05	0.05	.34	-0.16	0.06			.71		
	Working Memory (MTP)	-0.30	0.11	.007	-0.51	-0.08			.72		
	Visuospatial Memory (MTS)	-0.21	0.07	.003	-0.34	-0.07	-0.19	0.06	.001	-0.30	-0.08

B = Coefficient from the linear regression; SE(B) = Standard error of B; CI = Confidence Interval; R_{BV} = R from bootstrap validation
Multivariate models selected using a stepwise algorithm (p<.05 to enter, >.10 to exit)
Education was not always available in theatre so officer vs. enlisted was used as a surrogate.

© 2019 Mac Donald CL et al. *JAMA Netw Open*.

eTable 7. Linear Regression
Prediction of 5-Year PTSD Symptom Severity

0-7 Day Predictors		Univariate					Multivariate R = 0.55, R_{BV} = 0.36				
		B	SE(B)	p-value	95% CI for B		B	SE(B)	p-value	95% CI for B	
Demographics & 0-7 Day Outcomes	Age in Combat	-1.03	0.48	.03	-1.98	-0.08			.39		
	Officer vs. Enlisted	-26.65	7.19	<.001	-40.94	-12.36			.05		
	Female vs. Male	-16.74	8.61	.06	-33.86	0.38			.95		
	Branch of Service	17.97	5.96	.003	6.12	29.81			.69		
	TBI Diagnosis	28.18	5.41	<.001	17.43	38.92	17.59	6.30	.006	5.07	30.12
	Number of Deployments	0.88	2.03	.66	-3.15	4.92			.87		
	Number of Subsequent Concussions	7.02	3.32	.04	0.41	13.62			.37		
	Concussion Symptoms (RPCSQ)	1.11	0.21	<.001	0.69	1.52	0.72	0.24	.004	0.24	1.20
	PTSD Symptom Severity (PCL-M)	1.07	0.22	<.001	0.64	1.51			.31		
	Depression Symptom Severity (BDI)	1.66	0.41	<.001	0.85	2.47			.38		
	Combat Intensity (CES)	1.17	0.27	<.001	0.64	1.70			.37		
	Postural Stability (BESS)	0.04	0.40	.92	-0.76	0.84			.27		
0-7 Day ANAM Testing	Sleep Impairment	7.90	2.30	.001	3.34	12.46			.95		
	Simple Reaction Time	0.03	0.03	.27	-0.03	0.09			.31		
	Simple Reaction Time -R	0.03	0.02	.17	-0.01	0.07			.32		
	Processing Speed (PRT)	-0.21	0.18	.25	-0.58	0.15			.26		
	Associative Learning (CSL)	-0.14	0.23	.55	-0.59	0.32			.32		
	Delayed Memory (CSD)	-0.31	0.21	.14	-0.72	0.11			.83		
	Working Memory (MTP)	-0.97	0.42	.02	-1.80	-0.13			.69		
	Visuospatial Memory (MTS)	-0.29	0.27	.28	-0.83	0.25			.71		

B = Coefficient from the linear regression; SE(B) = Standard error of B; CI = Confidence Interval; R_{BV} = R from bootstrap validation
Multivariate models selected using a stepwise algorithm (p<.05 to enter, >.10 to exit)
Education was not always available in theatre so officer vs. enlisted was used as a surrogate.

© 2019 Mac Donald CL et al. *JAMA Netw Open*.

eTable 8. Linear Regression<br>Prediction of 5-Year Cognitive Performance											
0-7 Day Predictors		Univariate					Multivariate R = 0.55, R_{BV} = 0.34				
		B	SE(B)	p-value	95% CI for B		B	SE(B)	p-value	95% CI for B	
Demographics & 0-7 Day Outcomes	Age in Combat	-0.36	0.16	.03	-0.68	-0.04			.68		
	Officer vs. Enlisted	-6.08	2.43	.01	-10.91	-1.26			.50		
	Female vs. Male	-5.50	2.80	.05	-11.07	0.06			.89		
	Branch of Service	5.62	1.96	.005	1.72	9.51			.58		
	TBI Diagnosis	7.70	1.84	<.001	4.03	11.36	5.35	1.94	.007	1.49	9.20
	Number of Deployments	0.25	0.86	.77	-1.45	1.95			.56		
	Number of Subsequent Concussions	1.19	1.10	.28	-1.00	3.38			.76		
	Concussion Symptoms (RPCSQ)	0.14	0.08	.06	-0.01	0.30			.74		
	PTSD Symptom Severity (PCL-M)	0.17	0.08	.03	0.02	0.33			.80		
	Depression Symptom Severity (BDI)	0.14	0.14	.34	-0.15	0.42			.92		
	Combat Intensity (CES)	0.19	0.10	.05	0.00	0.38			.86		
	Postural Stability (BESS)	-0.18	0.13	.16	-0.44	0.07	-0.27	0.11	.02	-0.50	-0.05
0-7 Day ANAM Testing	Sleep Impairment	1.85	0.77	.02	0.32	3.38			.82		
	Simple Reaction Time	0.01	0.01	.48	-0.01	0.03			.42		
	Simple Reaction Time -R	0.01	0.01	.29	-0.01	0.02			.59		
	Processing Speed (PRT)	-0.16	0.06	.006	-0.28	-0.05			.17		
	Associative Learning (CSL)	-0.16	0.07	.04	-0.30	-0.01			.47		
	Delayed Memory (CSD)	-0.14	0.07	.04	-0.28	-0.01			.20		
	Working Memory (MTP)	-0.58	0.13	<.001	-0.83	-0.33	-0.45	0.13	.001	-0.72	-0.18
	Visuospatial Memory (MTS)	-0.19	0.09	.03	-0.36	-0.02			.41		

B = Coefficient from the linear regression; SE(B) = Standard error of B; CI = Confidence Interval; R_{BV} = R from bootstrap validation
Multivariate models selected using a stepwise algorithm (p<.05 to enter, >.10 to exit)
Education was not always available in theatre so officer vs. enlisted was used as a surrogate.

© 2019 Mac Donald CL et al. *JAMA Netw Open*.

eTable 9 is provided in Panels A, B, and C for clarity given the large number of parameters for each optimization analysis.

Multivariate models selected using a stepwise algorithm (p<.05 to enter, >.10 to exit). Education was not always available in theatre so officer vs. enlisted was used as a surrogate. Given that 5-year impairment was primarily observed in domains of neurobehavior and mental health and not cognitive dysfunction, acute assessments for these domains were focused on for this optimization process. This included the RPCSQ for Concussion Symptoms, PCL-M for PTSD symptoms, BDI for depression symptoms. While all 3 measures provided comparable predictive ability, the PCL-M edged out slightly from the others as best predicting all three primary domains of long-term outcome (global disability, neurobehavioral impairment, PTSD symptom severity.

For each Table B = Coefficient from the logistic regression; SE(B) = Standard error of B; OR = Odds ratio; CI = Confidence Interval

eTable 9A. Prediction Modeling Optimization of a Single Acute Predictor for Multidomain 5-Year Outcome - Global Outcome

0-7 Day Predictors	Multivariate (R=0.61)						Multivariate (R=0.68)						Multivariate (R=0.58)					
	B	SE(B)	OR	p-value	95% CI for OR		B	SE(B)	OR	p-value	95% CI for OR		B	SE(B)	OR	p-value	95% CI for OR	
Age in Combat				0.34						0.27						0.28		
Officer vs. Enlisted				0.13						0.08						0.07		
Female vs. Male				0.4						0.44						0.43		
Branch of Service				0.96						0.73						0.83		
TBI Diagnosis	2.3	0.67	9.7	0.001	2.64	35.84	2.5	0.65	13	< .001	3.49	44.38	2.8	0.65	17	< .001	4.62	59.05
Number of Deployments				0.42						0.57						0.45		
Number of Subsequent Concussions				0.09						0.09						0.11		
Concussion Symptoms (RPCSQ)	0.1	0.03	1.1	0.01	1.01	1.12												
PTSD Symptom Severity (PCL-M)							0.1	0.03	1.1	0.02	1.01	1.13						
Depression Symptom Severity (BDI)													0.1	0.05	1.1	0.02	1.02	1.22

© 2019 Mac Donald CL et al. *JAMA Netw Open*.

eTable 9B. Prediction Modeling Optimization of a Single Acute Predictor for Multidomain 5-Year Outcome - Neurobehavior Impairment

0-7 Day Predictors	Multivariate (R=0.65)					Multivariate (R=0.68)					Multivariate (R=0.69)				
	B	SE(B)	p-value	95% CI for B		B	SE(B)	p-value	95% CI for B		B	SE(B)	p-value	95% CI for B	
Age in Combat			0.74					0.6					0.7		
Officer vs. Enlisted	-4.2	1.7	0.02	-7.55	-0.79	-4.22	1.7	0.01	-7.5	-0.93	-4.2	1.64	0.01	-7.5	-0.9
Female vs. Male			0.48					0.30					0.3		
Branch of Service			0.48					0.32					0.3		
TBI Diagnosis	8.11	1.37	< .001	5.39	10.83	6.74	1.46	< .001	3.84	9.64	7.13	1.37	< .001	4.4	9.86
Number of Deployments			0.8					0.89					0.99		
Number of Subsequent Concussions			0.17					0.33					0.4		
Concussion Symptoms (RPCSQ)			0.05												
PTSD Symptom Severity (PCL-M)						0.14	0.06	0.02	0.02	0.25					
Depression Symptom Severity (BDI)											0.26	0.1	0.01	0.06	0.46

eTable 9C. Prediction Modeling Optimization of a Single Acute Predictor for Multidomain 5-Year Outcome – PTSD Severity

0-7 Day Predictors	Multivariate (R=0.54)					Multivariate (R=0.58)					Multivariate (R=0.58)				
	B	SE(B)	p-value	95% CI for B		B	SE(B)	p-value	95% CI for B		B	SE(B)	p-value	95% CI for B	
Age in Combat			0.36					0.73					0.82		
Officer vs. Enlisted			0.07			-14.34	7.1	0.05	-28.4	-0.28	-14.21	7.06	0.05	-28.3	-0.2
Female vs. Male			0.8					0.28					0.26		
Branch of Service			0.56					0.73					0.64		
TBI Diagnosis	17.8	6.45	0.007	5	30.67	16.1	6.2	0.01	3.72	28.47	18.66	5.9	0.002	6.93	30.4
Number of Deployments			0.84					0.77					0.65		
Number of Subsequent Concussions			0.36					0.59					0.58		
Concussion Symptoms (RPCSQ)	0.71	0.26	0.007	0.2	1.22										
PTSD Symptom Severity (PCL-M)						0.73	0.25	0.005	0.23	1.23					
Depression Symptom Severity (BDI)											1.25	0.43	0.004	0.4	2.09

eREFERENCES

1. King NS, Crawford S, Wenden FJ, Moss NE, Wade DT. The Rivermead Post Concussion Symptoms Questionnaire: a measure of symptoms commonly experienced after head injury and its reliability. *Journal of neurology.* 1995;242(9):587-592.
2. Yeager DE, Magruder KM, Knapp RG, Nicholas JS, Frueh BC. Performance characteristics of the posttraumatic stress disorder checklist and SPAN in Veterans Affairs primary care settings. *General hospital psychiatry.* 2007;29(4):294-301.
3. Homaifar BY, Brenner LA, Gutierrez PM, et al. Sensitivity and specificity of the Beck Depression Inventory-II in persons with traumatic brain injury. *Archives of physical medicine and rehabilitation.* 2009;90(4):652-656.
4. Keane T, Fairbank J, Caddell J, Zimering R, Taylor K, Mora C. Clinical evaluation of a measure to assess combat exposure. *Psychological Assessment.* 1989(1):53-55.
5. Guskiewicz KM, Ross SE, Marshall SW. Postural Stability and Neuropsychological Deficits After Concussion in Collegiate Athletes. *Journal of athletic training.* 2001;36(3):263-273.
6. Cernich A, Reeves D, Sun W, Bleiberg J. Automated Neuropsychological Assessment Metrics sports medicine battery. *Archives of clinical neuropsychology : the official journal of the National Academy of Neuropsychologists.* 2007;22 Suppl 1:S101-114.
7. Tombough T. The Test of Memory Malingering. *Multi-Health Systems.* 1996.
8. Wilson JT, Pettigrew LE, Teasdale GM. Structured interviews for the Glasgow Outcome Scale and the extended Glasgow Outcome Scale: guidelines for their use. *Journal of neurotrauma.* 1998;15(8):573-585.
9. Conners C, Staff. M. *Conners' Continuous Performance Test II: Computer program for Windows technical guide and software manual.* North Tonwanda, NY: Multi-Health Systems; 2000.
10. Delis D, Kramer J, Kaplan E, B O. *California Verbal Learning Test Manual: Second Edition, Adult Version.* San Antonio, Tx: Psychological Corporation; 2000.
11. Matthews C, Kløve H. *Instruction manual for the Adult Neuropsychology Test Battery.* Madison, WI: University of Wisconsin Medical School; 1964.
12. Reitan R. *Trail Making Test manual for administration and scoring.* Tuscon, AZ: Reitan Neuropsychology Laboratory; 1992.
13. Benton A, Hamsher K, A S. *Multilingual Aphasia Examination (3rd ed.).* Iowa City, Ia: AJA Associates; 1983.
14. Wechsler D. *Wechsler Test of Adult Reading (WTAR) Manual.* New York: Psychological Corporation; 2001.
15. Bechara A, Damasio AR, Damasio H, Anderson SW. Insensitivity to future consequences following damage to human prefrontal cortex. *Cognition.* 1994;50(1-3):7-15.
16. Delis DC, Kaplan, E. & Kramer, J.H. *Delis-Kaplan Executive Function System (D-KEFS): Examiner's manual.* San Antonio, TX: The Psychological Corporation; 2001.
17. Ruff R, Light R, Parker S. Visuospatial learning: Ruff Light Trail Learning Test. *Archives of clinical neuropsychology : the official journal of the National Academy of Neuropsychologists.* 1996;11(4):313-327.

© 2019 Mac Donald CL et al. *JAMA Netw Open*.
